# Revascularization During Cardiac Arrest While Receiving Extracorporeal Life Support in Patients With Acute Myocardial Infarction

**DOI:** 10.1016/j.jacadv.2024.101455

**Published:** 2024-12-13

**Authors:** Takahiro Nakashima, Marina Arai, Akihiko Inoue, Toru Hifumi, Tetsuya Sakamoto, Yasuhiro Kuroda, Yoshio Tahara, SAVE-J II Group

**Affiliations:** a Department of Emergency Medicine and The Harry Max Weil Institute for Critical Care Research and InnovationUniversity of Michigan, Ann Arbor, Michigan, USA; b Department of Preventive Medicine and Epidemiologic Informatics, National Cerebral and Cardiovascular Centre, Suita, Japan; c Department of Cardiovascular Medicine, Graduate School of Medicine, Tohoku University, Sendai, Japan; d Department of Emergency and Critical Care Medicine, Hyogo Emergency Medical Center, Kobe, Japan; e Department of Emergency and Critical Care Medicine, St. Luke's International Hospital, Tokyo, Japan; f Department of Emergency Medicine, Showa General Hospital, Tokyo, Japan; g Department of Emergency, Disaster and Critical Care Medicine, Kagawa University Hospital, Kagawa, Japan; h Department of Cardiovascular Medicine, National Cerebral and Cardiovascular Centre, Suita, Japan

**Keywords:** cardiac arrest, extracorporeal cardiopulmonary resuscitation, extracorporeal life support, percutaneous coronary intervention, return of spontaneous circulation

## Abstract

**Background:**

Extracorporeal cardiopulmonary resuscitation (ECPR) has allowed patients with refractory out-of-hospital cardiac arrest (OHCA) due to acute myocardial infarction (AMI) to receive primary percutaneous coronary intervention (PCI); they were previously ineligible.

**Objectives:**

The purpose of this study was to clarify the characteristics and outcomes of patients with OHCA secondary to AMI who underwent primary PCI during refractory cardiac arrest despite ECPR.

**Methods:**

Patients with AMI and OHCA aged ≥18 years who underwent PCI with ECPR in 2013 to 2018 were identified from a multicenter ECPR registry in Japan. The primary outcome was in-hospital mortality. We also assessed possible predictors of survival to discharge using mixed effects logistic regression to account for group differences among facilities.

**Results:**

Among 671 patients with AMI and OHCA who underwent PCI with ECPR from 30 institutions, 251 (37%) patients had refractory cardiac arrest despite ECPR initiation and subsequently underwent primary PCI. Following coronary reperfusion, 64.9% (163/251) of patients achieved the sustained return of spontaneous circulation (ROSC), 21.1% (53/251) survived, and 10.4% (26/251) had favorable neurological status at hospital discharge. Multivariable analysis revealed that intermittent prehospital ROSC (OR: 5.22; 95% CI: 1.54-17.79), shorter time to ECPR initiation (OR: 0.89; 95% CI: 0.82-0.98), and postprocedural TIMI flow grade 3 (OR: 5.08; 95% CI: 1.50-17.22) are significantly associated with survival to hospital discharge.

**Conclusions:**

Among patients with AMI and refractory OHCA treated with ECPR, one-third did not have sustained ROSC prior to PCI. Of those, two-thirds achieved sustained ROSC following reperfusion and one-fifth survived to discharge.

Acute myocardial infarction (AMI) is a leading cause of out-of-hospital cardiac arrest (OHCA).^1^^,^^2^ The priority for patients with AMI is early reperfusion therapy to minimize myocardial injury. International Liaison Committee on Resuscitation (ILCOR) and European Society of Cardiology guidelines recommend immediate coronary angiography (CAG) and primary percutaneous coronary intervention (PCI) in patients with OHCA who have return of spontaneous circulation (ROSC) and persistent ST-segment elevation on electrocardiography (ECG).^3^^,^^4^

There is accumulating evidence to support extracorporeal cardiopulmonary resuscitation (ECPR) as a bridge to fundamental treatment to address the etiology of OHCA in patients with refractory OHCA.^5^, ^6^, ^7^, ^8^ In recent years, the widespread adoption of ECPR has allowed patients with refractory OHCA to receive primary PCI with extracorporeal life support (ECLS); they had previously been ineligible for primary PCI. Such patients can achieve ROSC with coronary reperfusion. However, data on PCI for patients who do not achieve ROSC despite ECPR are limited.^9^^,^^10^ Understanding the characteristics and prognosis of those patients could help improve reperfusion strategies in patients with refractory OHCA secondary to AMI. Therefore, we evaluated the prevalence and survival to hospital discharge of patients with OHCA secondary to AMI who underwent primary PCI during refractory cardiac arrest despite ECPR, using a nationwide multicenter registry of ECPR in Japan.

## Methods

### Study design

This is a retrospective analysis of data from the SAVE-J II (Study of Advanced Life Support for Ventricular Fibrillation with Extracorporeal Circulation in Japan), a multicenter database of patients treated with ECPR in Japan.^11^ The study was preregistered in the University Hospital Medical Information Network Clinical Trials Registry, the clinical trial registry in Japan (UMIN000036490). This study was approved by the Institutional Review Boards of Kagawa University (2018-110) and the National Cerebral and Cardiovascular Center (R20032-2). The requirement for patient consent was waived due to the retrospective nature of this study. This study followed the Strengthening the Reporting of Observational Studies in Epidemiology (STROBE) reporting guidelines.^12^

### SAVE-J II data collection and quality control

The SAVE-J II Registry has 36 participating institutions and 2,157 consecutive patients with OHCA aged ≥18 years admitted to the emergency department and treated with ECPR between January 1, 2013, and December 31, 2018 (Supplemental Appendix). Patients who achieved ROSC at the initiation of ECLS and those who declined participation through their family or other agents were excluded from the registry. ECPR data were collected using a standardized form in Research Electronic Data Capture (REDCap) that included age, sex, pre-existing medical conditions, medications, location of arrest, whether the arrest was witnessed by a bystander, whether bystander cardiopulmonary resuscitation (CPR) was performed, initial documented cardiac arrest rhythm, whether public access defibrillation was performed, etiology of cardiac arrest, whether the patient had intermittent ROSC before hospital arrival, initiation of ECLS or primary PCI, location of cannulation for ECLS, time interval between emergency call and hospital arrival, time when ECLS flow was initiated, cause of death, and Cerebral Performance Category (CPC) at hospital discharge. Primary diagnosis was classified as AMI, arrhythmia, myopathy, myocarditis, other cardiac cause, pulmonary embolism, or other noncardiac cause. AMI was deﬁned according to the third universal deﬁnition of myocardial infarction, which includes the detection of a rise in cardiac biomarkers and the identification of an intracoronary thrombus.^13^ ROSC was deemed to have occurred when there were confirmed spontaneous palpable pulsations for at least 60 consecutive seconds. Once ROSC was achieved, if CPR was required again due to recurrence of arrest, ROSC was considered intermittent. ROSC after ECLS initiation was determined by the presence of an organized ECG rhythm and either an arterial line waveform or spontaneous heartbeat. The degree of cardiac contraction and pulsation was not considered, as cardiac function recovery is typically insufficient in the early phase. For patients diagnosed with AMI, if the facility had a standardized reporting system for catheterization characteristics, information on CAG features and procedures was also collected, which included culprit vessel, number of vessels with coronary artery disease (CAD), presence of chronic total occlusion in a non–infarct-related artery, whether primary PCI was performed, whether an inotrope or vasopressor (noradrenaline, dopamine, or dobutamine) was used, and whether an intra-aortic balloon pump (IABP) was inserted.^14^ Initial and postprocedural coronary blood flow in the culprit vessel was quantified using the TIMI flow grading system.^15^ A coronary artery was considered a culprit vessel on the basis of CAG findings. The decision to perform CAG, final diagnosis of AMI, and decision to perform PCI were at the discretion of the treating clinician. The data set was limited to nonidentifiable elements. All participating centers received approval for this study from their local Institutional Review Board. The characteristics of the participating institutions have been described previously.^16^

### Management of patients with out-of-hospital cardiac arrest in Japan

In Japan, municipally governed fire stations are equipped with dispatch centers and operate 24 hours every day, providing uniform guideline-based resuscitation in accordance with the 2010 and 2015 ILCOR guidelines during the study period from 2013 to 2018. Paramedics are instructed to transport patients with OHCA to the nearest emergency medical center in the region. According to each institution’s protocol, patients with refractory OHCA who have no obvious nontraumatic cause of arrest are either directly transported to the cardiac catheterization laboratory (CCL) for ECLS cannula placement under fluoroscopic guidance or to the emergency department where an ECLS cannula is placed under ultrasound guidance. During the study period, numerous emergency centers incorporated emergency CAG as a standard procedure following the initiation of ECLS into their institutional protocols if a cardiac etiology was suspected or the probability of survival was deemed to be high by the treating clinician. The addition of IABP to ECPR is not mandatory and is typically used depending on the cause of cardiac arrest, with the aim of left ventricular unloading. Targeted temperature management was delivered according to the 2010 and 2015 guidelines.

### Inclusion and exclusion criteria

Of the 36 institutions participating in the SAVE-J II Registry, 6 institutions were excluded from this analysis due to lack of consistent catheterization reports in 50% or more cases. Participants from 30 institutions aged ≥18 years who received ECPR due to refractory OHCA prior to intensive care unit (ICU) admission were eligible for study inclusion. Patients were included in the analysis if they were diagnosed with AMI on the basis of CAG findings and underwent primary PCI for the culprit lesion. Patients with no outcome data were excluded.

### Outcome definitions

The primary outcome was survival to hospital discharge. The secondary outcomes of interest were survival with favorable neurological status at hospital discharge, defined as CPC 1 (full recovery or mild disability) or CPC 2 (moderate cerebral disability) after cardiac arrest, and successful weaning from ECLS.^17^

### Statistical analysis

For baseline patient characteristics and outcomes, results are presented as medians and IQR, or n (percentage). Continuous variables were compared using the Mann–Whitney U test. Categorical variables were compared using the chi-squared test. The cumulative probability of in-hospital survival through 30 and 180 days was calculated using Kaplan-Meier methods (log-rank test) and presented with 95% CI. Patients were administratively censored if discharged prior to reported time point. The ORs with 95% CIs of primary and secondary outcomes were assessed using mixed effects logistic regression to account for group differences between facilities because a comprehensive bundle of postresuscitation care could be based on institutional practice rather than on a case-by-case basis. Adjustment was performed on the basis of the following clinical characteristics: age, sex, location of cardiac arrest (residence or not), witnessed status, bystander CPR status, initial documented rhythm (shockable or nonshockable rhythm), intermittent prehospital ROSC, location of cannulation for ECLS (CCL or not), and time interval from emergency call to ECLS initiation.

To identify possible predictors of survival to discharge in patients who did not achieve ROSC prior to primary PCI, we performed univariable and multivariable analyses. Univariable models included clinical characteristics, CAG findings, or procedures before ICU admission and facility as independent variables. All variables reaching *P* < 0.10 in a univariable analysis were included in the multivariable model.

A *P* value <0.05 was considered statistically significant. Statistical analyses were performed with R statistical software, version 4.2.3 (R Foundation for Statistical Computing). We performed multivariable imputation for missing data by chained equations.

## Results

**Figure 1:**
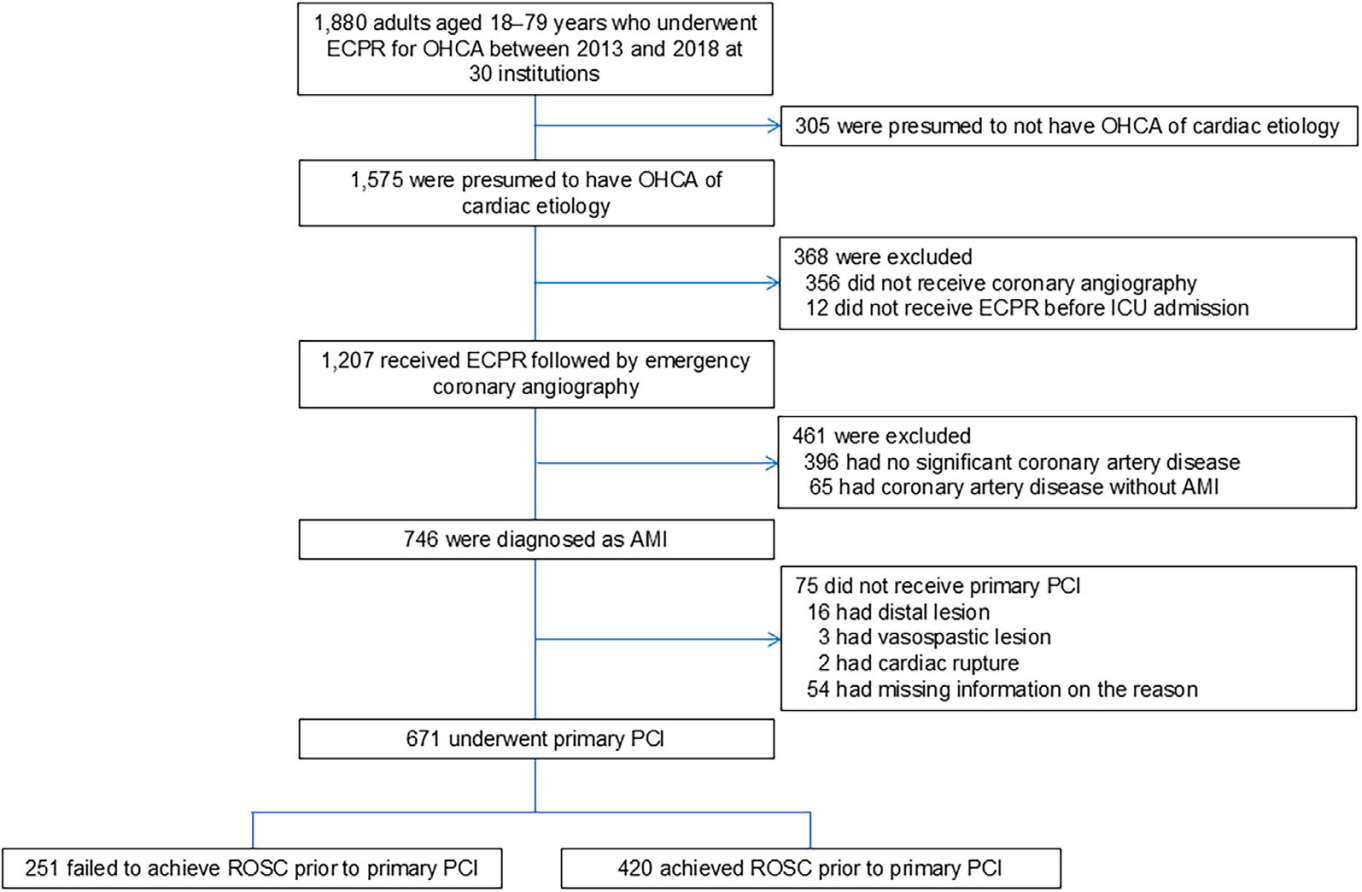
**Study Flow** AMI = acute myocardial infarction; ECPR = extracorporeal cardiopulmonary resuscitation; ICU = intensive care unit; OHCA = out-of-hospital cardiac arrest; PCI = percutaneous coronary intervention; ROSC = return of spontaneous circulation.

From January 1, 2013, to December 31, 2018, a total of 1,880 patients aged 18 to 79 years underwent ECPR due to OHCA across 30 institutions (Figure 1). Of these, 1,207 patients underwent emergency CAG. According to angiographic criteria, AMI was considered the cause of OHCA in 746 patients. Excluding 76 patients who did not undergo primary PCI, 671 patients underwent primary PCI on a culprit lesion. Despite the initiation of ECLS, 251 (37.4%) patients had sustained cardiac arrest and subsequently underwent primary PCI. The remaining 420 (62.6%) patients achieved ROSC before primary PCI.

Table 1 shows the clinical characteristics of the study patients. Patients with OHCA secondary to AMI who had refractory cardiac arrest despite ECPR initiation were more likely to be younger (median [Q1-Q3]: 61 [51-67 ] vs 63 [54-69] years), have a lower prevalence of intermittent prehospital ROSC (6.8% [17/251] vs 16.0% [67/420]), longer duration from emergency call to hospital arrival (median [Q1-Q3]: 33 [26-42] vs 30 [24-37] minutes), and receive cannulation for ECLS in CCL (55.4% [139/251] vs 43.3% [182/420]), compared with those with ROSC prior to primary PCI. There were no differences in witnessed status, bystander CPR status, initial documented rhythm, or automated external defibrillator attempt between the groups. The percentages of missing values are shown in Supplemental Table 1.

**Table 1:** Clinical Characteristics of Patients With AMI and OHCA Who Received ECPR

	No ROSC Prior to PCI			ROSC Followed by PCI (n = 420)		
	Total (n = 251)	Survive (n = 53)	Death (n = 198)		*P* Value^a^	*P* Value^b^
Age, y	61 (51, 67)	56 (50, 66)	62 (52, 67)	63 (54, 69)	0.011	0.16
Men	224 (89.2)	46 (86.8)	182 (91.9)	375 (89.3)	0.67	0.38
Pre-existing medical condition^c^	175 (69.7)	37 (69.8)	141 (71.2)	299 (71.2)	0.70	0.60
Hypertension	93 (37.1)	22 (41.5)	71 (35.9)	152 (36.2)		
Diabetics	55 (21.9)	11 (20.8)	46 (23.2)	110 (26.2)		
Dyslipidemia	33 (13.1)	8 (15.1)	26 (13.1)	76 (18.1)		
Heart disease	56 (22.3)	3 (5.7)	53 (26.8)	88 (21.0)		
Cerebral disease	12 (4.8)	1 (1.9)	11 (5.6)	32 (7.6)		
Chronic kidney disease	11 (4.4)	2 (3.8)	10 (5.1)	25 (6.0)		
Medication use^d^	108 (43.0)	17 (32.1)	94 (47.5)	215 (51.2)	0.19	0.083
Antiplatelet	27 (10.8)	2 (3.8)	25 (12.6)	70 (16.7)		
Anticoagulant	11 (4.4)	0 (0)	12 (6.1)	9 (2.1)		
Beta-blocker	14 (5.6)	2 (3.8)	13 (6.6)	34 (8.1)		
ACE inhibitor	17 (6.8)	1 (1.9)	16 (8.1)	16 (3.8)		
ARB	14 (5.6)	1 (1.9)	15 (7.6)	41 (9.8)		
Location					0.44	0.39
Home	86 (34.3)	13 (24.5)	74 (37.3)	131 (31.2)		
Public place	41 (16.3)	12 (22.6)	30 (15.2)	82 (19.5)		
Street	41 (16.3)	11 (20.8)	31 (15.7)	71 (16.9)		
Workplace	34 (13.5)	5 (9.4)	29 (14.6)	40 (9.5)		
Clinic	2 (0.8)	1 (1.9)	1 (0.5)	1 (0.2)		
Ambulance	29 (11.6)	7 (13.2)	22 (11.1)	62 (14.8)		
Other	14 (5.6)	4 (7.5)	11 (5.6)	31 (7.4)		
Witness	196 (78.1)	44 (83.0)	155 (78.3)	340 (81.0)	0.80	0.093
Bystander CPR	138 (55.0)	30 (56.6)	110 (55.6)	253 (60.2)	0.50	0.14
Initial rhythm					0.43	0.034
VF	187 (74.5)	48 (90.6)	141 (71.2)	294 (70.0)		
pVT	5 (2.0)	0 (0)	5 (2.5)	9 (2.1)		
PEA	39 (15.5)	4 (7.5)	36 (18.2)	87 (20.7)		
Asystole	13 (5.2)	0 (0)	14 (7.1)	27 (6.4)		
AED use	177 (70.5)	39 (73.6)	138 (69.7)	284 (67.6)	0.51	0.70
Intermittent prehospital ROSC	17 (6.8)	9 (17.0)	8 (4.0)	67 (16.0)	0.001	0.003
Time interval between emergency call and ECLS initiation, min	56 (47, 68)	49 (39, 58)	57 (48, 70)	55 (45, 68)	0.67	<0.001
Time interval between emergency call to hospital arrival, min	33 (26, 42)	29 (24, 39)	34 (27, 44)	30 (24, 37)	0.004	0.023
Time interval between hospital arrival and ECLS initiation, min	20 (14, 30)	16 (12, 27)	22 (14, 31)	22 (15, 34)	0.042	0.007
Location of ECLS cannulation					0.003	0.76
CCL	139 (55.4)	32 (60.4)	111 (56.1)	182 (43.3)		
ED	107 (42.6)	21 (39.6)	86 (43.4)	236 (56.2)		
Others	1 (0.4)	0 (0)	1 (0.5)	0 (0)		

Values are medians (Q1, Q3) or n (%).

ACE = angiotensin-converting enzyme; AED = automated external defibrillator; AMI = acute myocardial infarction; ARB = angiotensin receptor blockers; CCL = cardiac catheterization laboratory; CPR = cardiopulmonary resuscitation; ECLS = extracorporeal life support; ECPR = extracorporeal cardiopulmonary resuscitation; ED = emergency department; OHCA = out-of-hospital cardiac arrest; PCI = percutaneous coronary intervention; PEA = pulseless electrical activity; pVT = pulseless ventricular tachycardia; ROSC = return of spontaneous circulation; VF = ventricular fibrillation.

a Comparison between no ROSC prior to PCI (total) vs ROSC followed by PCI.

b Comparison between survival vs death among no ROSC.

c Pre-existing medical conditions were defined as the presence of at least one of the following at hospital admission: hypertension, diabetes, dyslipidemia, heart disease, cerebrovascular disease, or chronic kidney disease.

d Medication use was defined as the presence of at least one of the following: antiplatelet agents, anticoagulants, beta-blockers, ACE inhibitors, or ARBs.

### Primary and secondary outcomes

**Figure 2:**
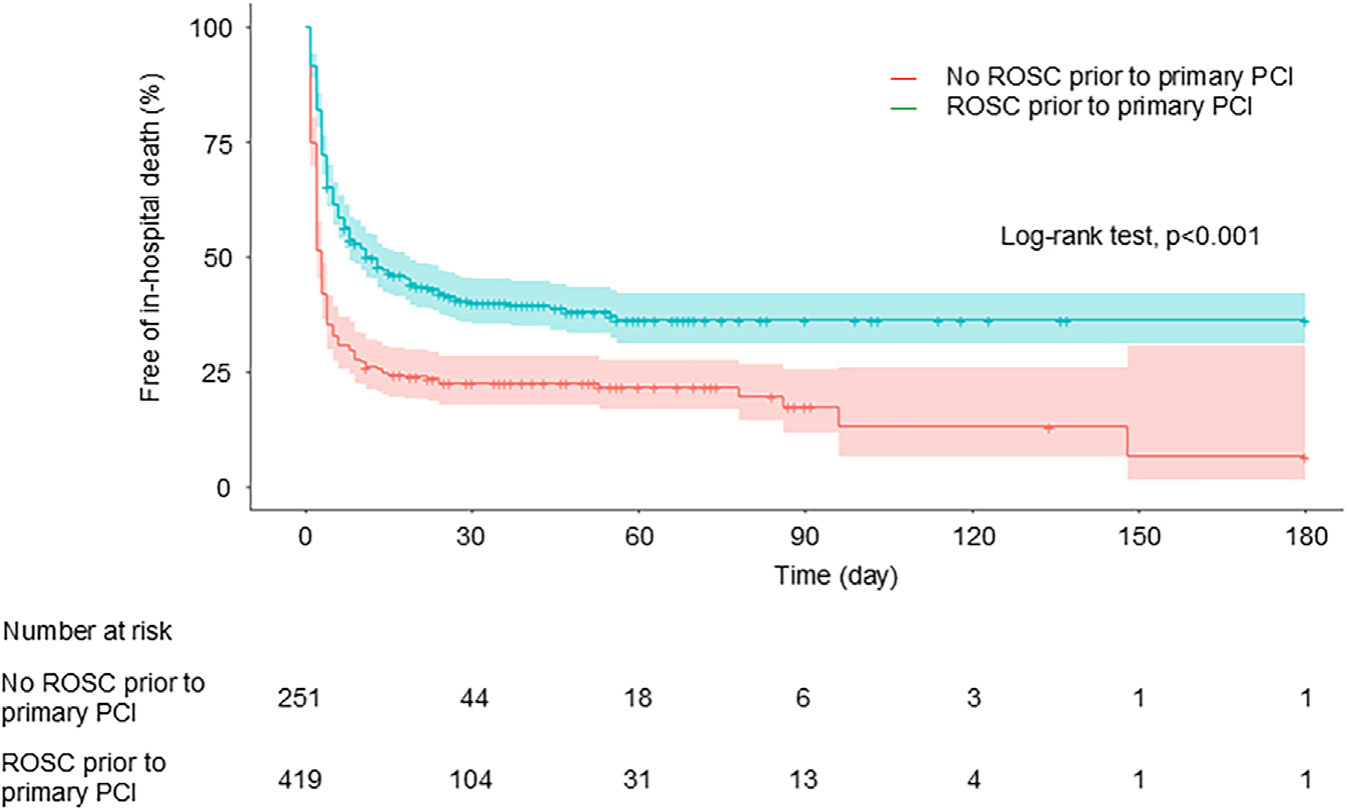
**Cumulative Probability of Survival in Hospital** Abbreviations as in Figure 1.

Following coronary reperfusion, 64.9% (163/251) of patients with refractory cardiac arrest despite ECLS initiation achieved ROSC. In 670 patients with available outcome data (≥99%), the Kaplan–Meier survival curve showed that estimated survival was 22.5% (95% CI: 16.9%-27.6%) at 30 days and 6.5% (95% CI: 1.4%-30.7%) at 180 days in patients who did not achieve ROSC prior to primary PCI whereas estimated survival was 40.0% (95% CI: 35.4%-45.2%) at 30 days and 36.3% (95% CI: 31.2%-42.1%) at 180 days in those who achieved ROSC followed by primary PCI, respectively (log-rank test, *P* < 0.001) (Figure 2). Among patients who did not achieve ROSC prior to primary PCI, 21.1% (53/251) survived to hospital discharge as the primary outcome (Table 2). For secondary outcomes, 10.4% (26/251) had favorable neurological status at hospital discharge, and 27.1% (68/251) were successfully weaned from ECLS. Meanwhile, in those who achieved ROSC followed by primary PCI, 40.6% (170/419) survived to discharge (*P* < 0.001). The percentage of favorable neurological status at hospital discharge and successful ECLS weaning was 22.2% (93/419) and 53.1% (223/420), respectively (all *P* < 0.001).

**Table 2:** Primary and Secondary Outcomes

			Multivariable^b^	
	No ROSC Prior to PCI	ROSC Followed by PCI	Adjusted OR (95% CI)	*P* Value
Primary outcome				
Survival at hospital discharge	53/251 (21.1)	170/419 (40.6)	0.36 (0.24-0.54)	<0.001
Secondary outcome				
Favorable neurological status at hospital discharge^a^	26/251 (10.4)	93/419 (22.2)	0.34 (0.20-0.57)	<0.001
Successful weaning from ECLS	68/251 (27.1)	223/420 (53.1)	0.29 (0.19-0.47)	<0.001

Values are n/N (%) unless otherwise indicated.

CPC = Cerebral Performance Category; other abbreviations as in Table 1.

a Favorable neurological status was defined as CPC 1 (full recovery or mild disability), or CPC 2 (moderate cerebral disability).

b Adjusted for age, sex, pre-existing medical condition, medications, cardiac arrest at home, witnessed status, bystander CPR, initial shockable rhythm, AED use, ECLS cannulation at CCL, time interval between onset and ECLS initiation, and intermittent prehospital ROSC, and accounted for interfacility differences.

### Patients who did not achieve ROSC prior to primary PCI

Focusing on patients with OHCA secondary to AMI who did not achieve ROSC prior to primary PCI (Table 1), patients with survival to hospital discharge had shorter time between emergency call and ECLS initiation and higher proportions of them had an initial shockable rhythm and intermittent prehospital ROSC, respectively, compared with those who died in the hospital. Table 3 shows the CAG features and procedures prior to ICU admission. Most patients had left anterior descending artery as the culprit vessel (60.4% [32/53] in the survival group vs 51.0% [101/198] in the death group) and single vessel disease (60.4% [32/53] vs 53.0% [105/198]). An inotrope was used in 39.6% (21/53) of patients in the survival group and 48.4% (96/198) of patients in the death group. A significantly higher proportion of the survival group had IABP use compared with the death group (98.1% [52/53] vs 87.4% [173/198], *P* = 0.043). Postprocedural TIMI flow grade 3 was significantly more common in the survival group than in the death group (86.8% [46/53] vs 65.2% [129/198], *P* = 0.021). The percentages of missing values are shown in Supplemental Table 2.

**Table 3:** Coronary Angiography Findings and Procedures Between Survival and Death Among Patients Without ROSC Prior to PCI

	Total (N = 251)	Survival (n = 53)	Death (n = 198)	*P* Value
Culprit vessel				0.55
Left main trunk artery	46 (18.3)	7 (13.2)	39 (19.7)	
Left anterior descending artery	133 (53.0)	32 (60.4	101 (51.0)	
Left circumflex artery	24 (9.6)	4 (7.5)	20 (10.1)	
Right coronary artery	44 (17.5)	10 (18.9)	34 (17.2)	
Number of vessels with coronary artery disease				0.47
1	137 (54.6)	32 (60.4)	105 (53.0)	
2	67 (26.7)	14 (26.4)	53 (26.8)	
3	47 (18.7)	7 (13.2)	40 (20.2)	
CTO in a noninfarct-related artery	65 (25.9)	12 (22.6)	53 (26.8)	
Initial TIMI flow grade before PCI				0.18
0	130 (51.8)	21 (39.6)	109 (55.1)	
1	59 (23.5)	16 (30.2)	43 (21.7)	
2	26 (10.4)	9 (17.0)	17 (8.6)	
3	13 (5.2)	2 (3.8)	11 (5.6)	
Postprocedural TIMI flow grade after PCI				0.021
0	7 (2.8)	0 (0)	7 (3.5)	
1	9 (3.6)	0 (0)	9 (4.5)	
2	40 (15.9)	3 (5.7)	37 (18.7)	
3	175 (69.7)	46 (86.8)	129 (65.2)	
Inotrope/Vasopressor	117 (46.6)	21 (39.6)	96 (48.5)	0.19
Norepinephrine	97 (38.6)	19 (35.8)	78 (39.4)	
Dopamine	17 (6.8)	3 (5.7)	14 (7.1)	
Dobutamine	33 (14.1)	7 (13.2)	26 (13.1)	
IABP use	225 (89.6)	52 (98.1)	173 (87.4)	0.043

Values are n (%).

CTO = chronic total occlusion; IABP = intra-aortic balloon pump; other abbreviations as in Table 1.

### Predictors of survival to discharge in patients who did not achieve ROSC prior to primary PCI

We assessed the factors associated with survival to hospital discharge (Table 4). In univariable analyses accounting for interfacility differences, medication, initial shockable rhythm, intermittent prehospital ROSC, time interval between emergency call and ECLS initiation, and postprocedural TIMI flow grade 3 were significantly associated with survival to hospital discharge. Multivariable analysis that included clinical characteristics, CAG features, and procedures prior to ICU admission revealed that the occurrence of intermittent prehospital ROSC (adjusted OR: 5.22; 95% CI: 1.54-17.79; *P* = 0.009), shorter time interval between emergency call and ECLS initiation (adjusted OR: 0.89; 95% CI: 0.82-0.98; *P* = 0.014), and postprocedural TIMI flow grade 3 (adjusted OR: 5.08; 95% CI; 1.50-17.22; *P* = 0.010) are significantly associated with survival to hospital discharge.

**Table 4:** Predictor of Survival at Hospital Discharge Among Patients Without ROSC Despite ECPR

	Univariable Model^a^		Multivariable Model^b^	
	Adjusted OR	*P* Value	Adjusted OR	*P* Value
Age, y	0.98 (0.95-1.01)	0.13		
Men	0.58 (0.22-1.49)	0.26		
Pre-existing medical condition	0.83 (0.42-1.64)	0.59		
Medication	0.51 (0.27-0.96)	0.037	0.56 (0.27-1.16)	0.12
OHCA at home	0.54 (0.27-1.08)	0.085	0.62 (0.28-1.37)	0.24
Witnessed status	1.48 (0.65-3.36)	0.36		
Bystander CPR	1.08 (0.58-2.01)	0.81		
Initial shockable rhythm	4.12 (1.41-12.00)	0.010	3.18 (0.99-10.21)	0.053
AED use	1.21 (0.61-2.39)	0.58		
Intermittent prehospital ROSC	4.86 (1.77-13.30)	0.002	5.23 (1.54-17.79)	0.009
Time interval between emergency call and ECLS initiation, per 3 min	0.89 (0.82-0.96)	0.002	0.89 (0.82-0.98)	0.014
ECLS cannulation at CCL	1.18 (0.64-2.19)	0.60		
Culprit lesion of left main trunk artery or left anterior descending artery	1.08 (0.54-2.14)	0.83		
Multivessel disease	0.74 (0.40-1.38)	0.35		
CTO	0.79 (0.39-1.61)	0.52		
Initial TIMI flow grade 0 or 1	1.11 (0.80-1.54)	0.52		
Postprocedural TIMI flow grade 3	4.91 (1.56-15.45)	0.007	5.08 (1.50-17.23)	0.010
Inotrope/vasopressor use	0.62 (0.33-1.15)	0.13		
IABP use	7.51 (0.99-56.80)	0.052	5.05 (0.62-41.08)	0.13

Abbreviations as in Tables 1 and 3.

a Adjusted for facilities.

b Adjusted for all covariates reaching *P* < 0.10 in an univariable model, including age, initial shockable rhythm, intermittent prehospital ROSC, time interval between emergency call and ECPR initiation, postprocedural TIMI flow grade, IABP use, and facilities.

## Discussion

Our analysis based on a multicenter ECPR registry in Japan demonstrated that 37% of patients with OHCA secondary to AMI who underwent primary PCI have refractory cardiac arrest even after ECLS initiation. Of those, 61% achieved ROSC after achieving coronary reperfusion, 21% survived to hospital discharge, and 10% had favorable neurological status at hospital discharge. Intermittent prehospital ROSC, shorter time interval between emergency call and ECLS initiation, and postprocedural TIMI 3 flow were significantly associated with survival at hospital discharge (Central Illustration).

**Central Illustration:**
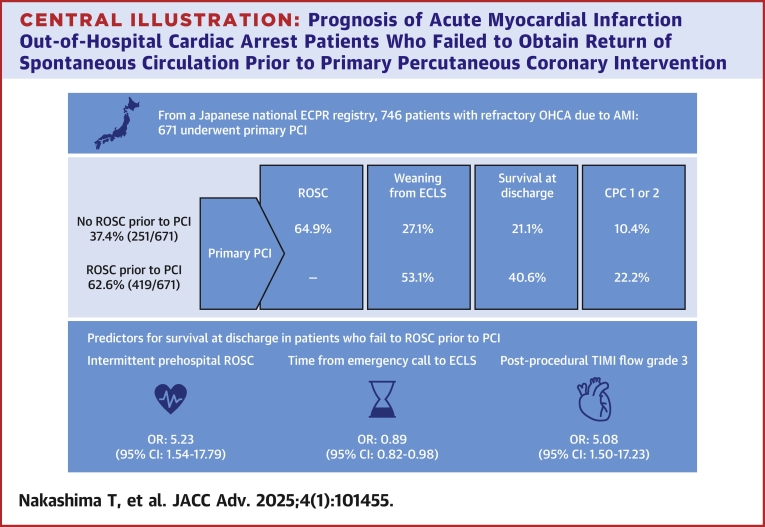
**Prognosis of Acute Myocardial Infarction Out-of-Hospital Cardiac Arrest Patients Who Failed to Obtain Return of Spontaneous Circulation Prior to Primary Percutaneous Coronary Intervention** ECLS = extracorporeal life support; CPC = Cerebral Performance Category; flow grade other abbreviations as in Figure 1.

The ECPR strategy makes time for advanced treatment in patients with refractory OHCA by maintaining organ perfusion. ECPR is not the definitive treatment for OHCA secondary to AMI; it is a bridge to primary PCI.^5^^,^^6^ The ARREST (Advanced R2Eperfusion STrategies for Refractory Cardiac Arrest) trial, a single-center randomized controlled trial (RCT) of 30 patients with OHCA of presumed cardiac etiology and a shockable rhythm, reported that 54% (7 of 13) of patients who received ECPR and CAG had a culprit vessel.^7^ The Prague OHCA study, a RCT of 256 patients with OHCA of presumed cardiac etiology, reported that 52% (64 of 123) of patients who underwent ECPR had acute coronary syndrome or CAD.^8^ In our study, 48% (760 of 1,574) of patients with OHCA of presumed cardiac etiology who underwent ECPR were diagnosed with AMI on the basis of CAG. The prevalence of AMI among patients treated with ECPR is similar to those in these RCTs from North America and Europe.^7^^,^^8^

According to ILCOR and European Society of Cardiology guidelines, CAG is indicated only in resuscitated patients with ST-segment elevation on post-ROSC ECG. Neither of these guidelines mention patients who do not achieve ROSC.^3^^,^^4^ However, the Parisian registry of OHCA patients, which enrolled 1,430 patients, reported that 29% of resuscitated patients without ST-elevation on post-ROSC ECG had a culprit lesion identified during immediate CAG and underwent primary PCI.^18^ In the ECPR era, many patients are able to undergo primary PCI under ECLS even if they have refractory cardiac arrest. Such patients, in certain instances, fail to achieve ROSC via ECLS flow, but they can achieve ROSC through coronary reperfusion. Consequently, discerning the optimal timing for coronary reperfusion, specifically, whether it should be performed during cardiac arrest or subsequent to the achievement of ROSC and ECG acquisition, poses a challenge. Yannopoulos et al reported that 84% of patients with ventricular fibrillation (VF) and OHCA had complex but treatable CAD, which is a valuable insight for decision-making for CAG and PCI.^6^ The INCEPTION (early INitiation of extraCorporeal lifE suPporT In refractory Out of hospital cardiac arrest) trial, involving 134 VF-OHCA patients, reported that 77% of participants had AMI as the cause of cardiac arrest.^19^ Our finding is consistent with their study, indicating VF/pulseless ventricular tachycardia as the most common cardiac arrest rhythm initially documented in patients with OHCA secondary to a coronary cause (74%).

Since ongoing myocardial ischemia induces refractory VF/pulseless ventricular tachycardia, coronary reperfusion promotes ROSC.^20^^,^^21^ Our study showed that 61% of patients with refractory cardiac arrest even after ECLS initiation achieved ROSC after coronary reperfusion. However, only 27% achieved weaning from ECLS and 10% survived to hospital discharge. Refractory cardiac arrest before coronary reperfusion might limit survival due to irreversible heart and brain injury. Our study also showed that existence of intermittent prehospital ROSC, shorter time between emergency call and ECLS initiation, and postprocedural TIMI flow grade 3 are associated with survival to hospital discharge. To enhance prompt access to ECPR, it is crucial to improve regional health care systems and institutional protocols.^22^^,^^23^ Single-center trials conducted in well-trained institutions showed that the time from collapse to ECLS initiation was 61 minutes,^7^^,^^8^ whereas it was 74 minutes in the multicenter INCEPTION trial.^19^ In addition, improving the PCI strategy, such as left ventricular unloading, is also effective for achieving successful PCI in patients with OHCA secondary to AMI.^14^^,^^24^^,^^25^ Our study raises new questions about CAG and PCI strategy for patients in the ECPR era who do not achieve ROSC after ECLS initiation. Further studies are needed to identify novel strategies to evaluate and increase the probability of neurologic recovery and survival for patients with OHCA due to AMI in the ECPR era.

Our study has several limitations. First, this study was not an RCT. Thus, there is the possibility of selection bias related to the quality of treatment. However, consecutive participants were enrolled with a high follow-up rate (≥99%) and we adjusted for potential selection bias. In addition, missing data were rare for most clinical variables (<5%) with the exception of medication (12%). Second, the SAVE-J II Registry has no detailed information on the devices used or door-to-balloon (D2B) time for primary PCI. The 2019 annual report from a Japanese PCI registry showed that a drug-eluting stent was deployed in 86% of patients with AMI and median D2B time was 70 (Q1-Q3: 54-90) minutes.^26^ Since in-hospital processes leading to primary PCI, which affects D2B time, could reflect institutional practices, we adjusted for interfacility differences. Finally, we were not able to evaluate the quality of conventional CPR before the initiation of ECLS flow.

## Conclusions

In a Japanese nationwide registry, among AMI patients who had refractory cardiac arrest after ECLS initiation, one-third did not achieve ROSC prior to PCI. Of these, two-thirds achieved ROSC after coronary reperfusion and one-fifth survived to discharge. Intermittent prehospital ROSC, prompt initiation of ECLS, and successful primary PCI are associated with survival to discharge.

Perspectives **COMPETENCY IN MEDICAL KNOWLEDGE:** In a multicenter ECPR registry in Japan, one-third of patients with AMI and OHCA underwent PCI during cardiac arrest under ECLS. Of those, one-fifth survived to discharge.

**TRANSLATIONAL OUTLOOK:** This study provides information on outcomes of patients with AMI-OHCA who underwent PCI during cardiac arrest despite ECLS initiation. Novel strategies are needed to evaluate and increase the probability of neurologic recovery and survival for patients with AMI-OHCA in the ECPR era.

## Funding support and author disclosures

Dr Hifumi has received a grant from the Asahi Kasei Japan unrelated to this work. This research was partially supported by a Grant-in-Aid for Scientific Research (C) (JP19K09419) from the Japan Society for the Promotion of Science (Dr. Nakashima). All other authors have reported that they have no relationships relevant to the contents of this paper to disclose.
