# Supplementary material for: Revascularization During Cardiac Arrest While Receiving Extracorporeal Life Support in Patients With Acute Myocardial Infarction
Source: JACC Adv. 2024 Dec 13;4(1):101455. doi: 10.1016/j.jacadv.2024.101455 (PMC11699304; doi:10.1016/j.jacadv.2024.101455)
Supplement: Supplementary material [file mmc1.docx]

**SUPPLEMENTARY MATERIAL**

**TABLE OF CONTENTS**

Supplemental Appendix**2**

SAVE-J II study group**2**

Supplemental Table**4**

Table S1. Percentage of missing values in the final analysis dataset**4**

Table S2. Percentage of missing values in the final analysis dataset**4**

**SAVE-J II study group**

Hirotaka Sawano, M.D., Ph.D. (Osaka Saiseikai Senri Hospital), Yuko Egawa, M.D., Shunichi Kato, M.D. (Saitama Red Cross Hospital), Kazuhiro Sugiyama, M.D., Maki Tanabe, M.D. (Tokyo Metropolitan Bokutoh Hospital), Naofumi Bunya, M.D., Takehiko Kasai, M.D. (Sapporo Medical University), Shinichi Ijuin, M.D., Shinichi Nakayama, M.D., Ph.D. (Hyogo Emergency Medical Center), Jun Kanda, M.D., Ph.D., Seiya Kanou, M.D. (Teikyo University Hospital), Toru Takiguchi, M.D., Shoji Yokobori, M.D., Ph.D. (Nippon Medical School), Hiroaki Takada, M.D., Kazushige Inoue, M.D. (National Hospital Organization Disaster Medical Center), Ichiro Takeuchi, M.D., Ph.D., Hiroshi Honzawa, M.D. (Yokohama City University Medical Center), Makoto Kobayashi, M.D., Ph.D., Tomohiro Hamagami, M.D. (Toyooka Public Hospital), Wataru Takayama, M.D., Yasuhiro Otomo, M.D., Ph.D. (Tokyo Medical and Dental University Hospital of Medicine), Kunihiko Maekawa, M.D. (Hokkaido University Hospital), Takafumi Shimizu, M.D., Satoshi Nara, M.D. (Teine Keijinkai Hospital), Michitaka Nasu, M.D., Kuniko Takahashi, M.D. (Urasoe General Hospital), Yoshihiro Hagiwara, M.D., M.P.H. (Imperial Foundation Saiseikai, Utsunomiya Hospital), Shigeki Kushimoto, M.D., Ph.D. (Tohoku University Graduate School of Medicine), Reo Fukuda, M.D. (Nippon Medical School Tama Nagayama Hospital), Takayuki Ogura, M.D., Ph.D. (Japan Red Cross Maebashi Hospital), Shin-ichiro Shiraishi, M.D. (Aizu Central Hospital), Ryosuke Zushi, M.D. (Osaka Mishima Emergency Critical Care Center), Norio Otani, M.D. (St. Luke’s International Hospital), Migaku Kikuchi, M.D., Ph.D. (Dokkyo Medical University), Kazuhiro Watanabe, M.D. (Nihon University Hospital), Takuo Nakagami, M.D. (Omihachiman Community Medical Center), Tomohisa Shoko, M.D., Ph.D. (Tokyo Women’s Medical University Medical Center East), Nobuya Kitamura, M.D., Ph.D. (Kimitsu Chuo Hospital), Takayuki Otani, M.D. (Hiroshima City Hiroshima Citizens Hospital), Yoshinori Matsuoka, M.D., Ph.D. (Kobe City Medical Center General Hospital), Makoto Aoki, M.D., Ph.D. (Gunma University Graduate School of Medicine), Masaaki Sakuraya, M.D., M.P.H. (JA Hiroshima General Hospital Hiroshima), Hideki Arimoto, M.D. (Osaka City General Hospital), Koichiro Homma, M.D., Ph.D. (Keio University School of Medicine), Hiromichi Naito, M.D., Ph.D. (Okayama University Hospital), Shunichiro Nakao, M.D., Ph.D. (Osaka University Graduate School of Medicine), Tomoya Okazaki, M.D., Ph.D. (Kagawa University Hospital), Yoshio Tahara, M.D., Ph.D. (National Cerebral and Cardiovascular Center), Hiroshi Okamoto, M.D, M.P.H. (St. Luke’s International Hospital), Jun Kunikata, M.D., Ph.D., Hideto Yokoi, M.D., Ph.D. (Kagawa University Hospital).

**Table S1. Missing values of clinical characteristics**

|  | **Total**  **(n=671)** |
| --- | --- |
| Age, n (%) | 1 (0.1) |
| Men, n (%) | 0 (0) |
| Pre-existing medical condition, n (%) | 32 (4.8) |
| Medication, n (%) | 80 (11.9) |
| Location, n (%) | 1 (0.1) |
| Witness, n (%) | 2 (0.3) |
| Bystander CPR, n (%) | 8 (1.1) |
| Initial Rhythm, n (%) | 5 (0.7) |
| AED use, n (%) | 0 (0) |
| Intermittent prehospital ROSC, n (%) |  |
| Time interval between emergency call to ECPR initiation, n (%) | 27 (4.0) |
| Time interval between emergency call to hospital arrival, n (%) | 13 (1.9) |
| Time interval between hospital arrival and ECPR initiation, n (%) | 17 (2.5) |
| Location of ECLS cannulation, n (%) | 1 (0.1) |

AED denotes automated external defibrillator; CPR, cardiopulmonary resuscitation; ECMO, extracorporeal membrane oxygenation; ECLS, extracorporeal life support; ECPR, extracorporeal cardiopulmonary resuscitation; ROSC, return of spontaneous circulation.

**Table S2. Missing values of coronary angiography and procedures**

|  | **Total**  **(n=251)** |
| --- | --- |
| Culprit vessel, n (%) | 4 (1.6) |
| Number of vessels with coronary artery disease, n (%) | 0 (0) |
| CTO in a non–infarct-related artery, n (%) | 2 (0.8) |
| Initial TIMI flow grade before PCI, n (%) | 23 (9.1) |
| Post-procedural TIMI flow grade after PCI, n (%) | 20 (7.9) |
| Inotrope / Vasopressor, n (%) | 18 (7.2) |
| IABP use, n (%) | 0 (0) |

CTO denotes chronic total occlusion; IABP, intra-aortic balloon pump; PCI, percutaneous coronary intervention; and TIMI, Thrombolysis in Myocardial Infarction.
